# Nanowires of polyaniline festooned silver coated paper electrodes for efficient solid-state symmetrical supercapacitors†

**DOI:** 10.1039/c8ra06784h

**Published:** 2018-10-02

**Authors:** A. Aashish, C. Molji, Ganesan Krishna Priya, Muthusamy Sankaran, Unnikrishnan Nair Saraswathy Hareesh, Sudha J. Devaki

**Affiliations:** Chemical Sciences and Technology Division, CSIR-National Institute for Interdisciplinary Science and Technology Trivandrum-695019 India sudhajd2001@yahoo.co.in; Materials Science and Technology Division, CSIR-National Institute for Interdisciplinary Science and Technology Trivandrum-695019 India; Academy of Scientific and Innovative Research (CSIR-NIIST Campus), CSIR-National Institute for Interdisciplinary Science and Technology Trivandrum-695019 India; ISRO Satellite Centre Old Airport Road, Vimanapura Post Bengaluru-560017 Karnataka India

## Abstract

This paper demonstrates a facile strategy for the development of nanosilver decorated polyaniline coated (PAg) paper-based electrodes for the fabrication of solid-state symmetrical supercapacitors. PAg based printing paper was developed through a two-step process involving initial silver nucleation and growth on the paper followed by aniline polymerization. The developed electrically conductive paper exhibited a highly porous structure and excellent mechanical stability. Further symmetrical supercapacitors having the configuration PAg/electrolyte/PAg were fabricated and evaluated for electrochemical performance such as specific capacitance (483 F g^−1^ and 613 F g^−1^ in aqueous 1 M H_2_SO_4_ and PVA–H_2_SO_4_ gel electrolytes respectively), energy density (69.56 and 85.13 W h kg^−1^), and power density (243.44 and 405.375 W kg^−1^) and cycling stability (90% of its capacitance retention even after 2000 cycles), exhibiting excellent performance under various bending conditions. All these exciting results suggest that the developed paper-based flexible solid-state energy device can serve as an efficient, sustainable, and low-cost energy storage system for portable microelectronic devices which are expected to revolutionize the perception of energy-storage devices in the electronics industry.

## Abstract 2

This paper demonstrates a facile strategy for the development of nanosilver decorated polyaniline coated (PAg) paper-based electrodes for the fabrication of solid-state symmetrical supercapacitors.

## Graphical abstract

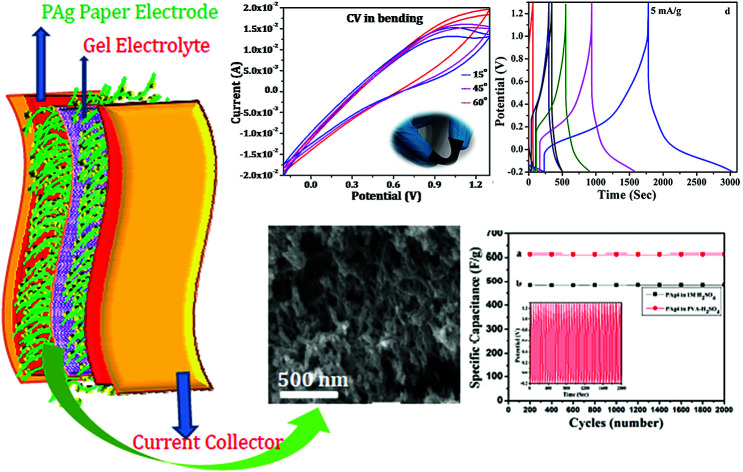

## 1. Introduction

The design and development of inexpensive, thin, flexible, light weight and even roll up portable electronic devices with multifunctional applications have recently received overwhelming interest among researchers from academia and industry. This warrants innovative technology for developing energy storage devices with less mass and flexibility endowed with high energy and power density even under bending conditions. Batteries and supercapacitors are the two important electrical energy storage systems where supercapacitors are superior to batteries in terms of life cycle, power density, environmental benignancy and safety.^1–6^

Various technologies such as microfabrication technology open up low-cost production for a high performing, robust, and adjustable micro supercapacitor system which further explores its feasibility to be integrated into miniaturized devices.^7^

Although the theoretical performance of these flexible energy storage devices is very high, their efficiency is extremely retarded owing to the assembly of various components in the device. This will greatly affect the commercialization of these portable electronic products.^8^ Hence intense care has to be taken for the development of efficient, flexible and high performance energy storage devices. Generally supercapacitors contain components such as electrodes, electrolytes, separators and current collectors. For the past two decades, enormous efforts have been made towards the development of nano-structured electrode materials. Qi Xue *et al.*, demonstrated high stability and promising capacitance of the boron nanowires in all alkaline, neutral, and acid aqueous electrolytes. The prepared nanostructures integrated on a carbon fiber cloth substrate achieved a capacitance up to 60.2 mF cm^−2^.^9^ These type of nanostructures can be integrated into the flexible substrates so as to enhance the properties through shortened diffusion length, superior specific surface area, enhance ion diffusion *etc*.^10,11^

In search of state-of-the art inexpensive, flexible, light weight, environmentally benign electrode materials, conductive polymer coated paper electrodes are receiving prominent scope of research interest among the scientific community.^12–16^ The conventional flexible electrodes such as PET-ITO/FTO, metal foils, possess major drawbacks such as its production using highly sophisticated instrumentation, high cost for the precursor materials, time consuming process for the deposition, above all they are easily oxidized which can cause considerable drop in the electrical conductivity and the poor adhesion between the active layer.^17–19^ Construction of paper-based supercapacitors with a reasonably good performance at a low price is highly beneficial, since paper is composed of large number of cellulose fibers/subnanofibrils with unique porous bulk structure along with rough and absorptive surface properties and high surface area.^20–22^ This may find advantageous as an electrode material for electrochemical systems. The main hindering thing is their high electrical surface resistivity of 10^15^ Ω sq^−1^ which limits their potential applications in the electronics industry. However, prior surface modifications in the form of coating or growing nanostructured materials are required to make them electronically conductive. In this aspect, conductive polymer composites can easily be infused in the cellulose network; further which can be made-up into diverse shapes and sizes for its usage as working electrode.^23–25^

Electrically conducting polymers such as polyaniline, polypyrrole is receiving interest since its conductivity can be tuned by controlling the reduction and oxidation process. The main draw back with conducting polymers based energy storage systems are its poor cycling stabilities and high self-discharge rates as well as mass transport limitation through the thick polymer mass. A promising strategy for solving this issue is either preparation of conducting polymers in nanoscale or depositing in a matrix with high surface area and porosity.^26–29^ L Yuan *et al.*, designed PANI paper based supercapacitor electrodes for self-powdered nanosystems. The electrodes were prepared using conventional three component system through the oxidation of aniline onto a conducting gold coated paper.^30^ An aerial capacitance of 50 mF cm^−2^ was reported. In another report by Cong *et al.*, developed graphene-PANI paper based electrode for supercapacitor through a two step strategy which measured maximum specific capacitance of 763 F g^−1^.^31^

Metal nanostructures such as copper, silver, gold are important materials for applications such as catalyst, optoelectronic devices due to their intrinsic properties which can be tailored with their nanosize and shape. Being highly conductive materials, silver has been the centre of attraction for the preparation of conductive nanomaterials. Silver nanoparticles have distinctive physicochemical and electrochemical properties including high chemical stability, catalytic activity *etc.* These properties make them of potential value in electronics, inks, and medical imaging *etc.* Several methods were reported for the preparation silver nanoparticles such as reduction of Ag^+^ using chemical methods, template aided methods; electrochemical methods, photo reduction, and so forth.^32,33^

Thus development of conductive polymer-silver nanocomposite is receiving immense interest which stems from their synergistic physicochemical and electrochemical properties. Especially polyaniline-silver nanocomposites have received significant attraction because of their excellent biocompatibility, electro-catalytic and electro-chemical properties.^34,35^ Several strategies are reported for the preparation of polyaniline-silver nanocomposite like dispersion of silver nanoparticles on the surface of the polymer, *in situ* polymerisation of conductive monomer around the silver nanoparticles and reduction of silver ions using monomer as a reducing agent.^36^ A report by Ajay Singh *et al.*, showed that free standing films of polypyrrole-silver nanocomposite films were used as electrode material for supercapacitors by photopolymerisation of pyrrole in presence of silver nitrate.^37^ From the aforementioned evidences, development of supercapacitors with excellent cycling stability, power and electron densities along with mechanical robustness and flexibility is receiving tremendous importance and subsequent fabrication of supercapacitor device of desired shape is therefore fascinating. So the present work envisages the preparation of conducting polyaniline decorated silver nanoparticle coated paper electrode and its characterization, demonstration as an efficient electrode for the fabrication of flexible and stable symmetrical supercapacitor.

## 2. Experimental section

### 2.1 Materials

Aniline, Ammonium persulphate (APS) and silver nitrate were purchased from Merck Limited, Mumbai, India. Polyvinylpyrrolidone (PVP) was purchased from Sigma Aldrich (Average M.W 10 000). All solvents used were analytical grade purchased from S.D fine chemicals. Printing paper was used for this experiment for electrode fabrication purpose.

### 2.2 Preparation of nanosilver decorated polyaniline coated paper

Silver nanoparticles were prepared by simple synthetic protocol as per the procedure given below.10 ml (in DMF) of 0.02 M silver nitrate was dissolved in 10 ml (DMF) of 0.1 M PVP which was used as capping agent to provide colloidal stability of the resulting nanoparticles. Paper strip was cut into 2 × 2 cm size and soaked into the above prepared Ag nanoparticle solution for 2 h. The colour of the paper changed to shining yellow and then brown revealing the formation of silver nanoparticle on the surface of the paper. The Ag coated paper was dried at 60 °C for 1 h under vacuum. Then polymerization of aniline was carried out on the Ag coated paper substrate.

Polyaniline was deposited on the silver coated paper through polymerization of aniline using APS as oxidant initiator at ice cold conditions. Silver coated paper strip was soaked in aniline monomer for 5, 15, 30, 60 minutes and gently taken out using a forceps to obtain uniform coating on varying the amount of aniline onto the paper. These samples are designated as PAg1, PAg2, PAg3, and PAg4 respectively. Then the aniline coated paper was introduced in the oxidant initiator which was kept at ice cold conditions. Gradually brown colour changed into blue and then green as the polymerization time increased which confirmed the formation of polarons (conductive charges) in polyaniline. Then the paper was removed from the chamber and washed several times with distilled water and then 1 M HCl. The conductivity of the films was optimized by the soaking time of aniline monomer and polymerization which is tabulated in Table 1. This silver decorated PANI coated papers were demonstrated for the fabrication of symmetrical supercapacitors.

**Table 1:** Electrical conductivity, amount of PANI deposited and coating thickness on the silver paper electrode

Sample	Conductivity (S cm^−1^)	Amount of PANI deposited (g)	Deposited coating thickness of PANI (mm)
PAg1	4.94	0.115	1.2
PAg2	39.32	0.125	1.75
PAg3	157.47	0.133	2.25
PAg4	211.243	0.15	3
PANI	82.44	0.126	1.65

### 2.3 Preparation of electrolytes

Aqueous (1 M H_2_SO_4_) and PVA–H_2_SO_4_ based electrolytes were prepared for the fabrication of supercapacitors. Both the electrolytes were dipped in Whatman filter paper which acts as separator. PVA–H_2_SO_4_ was prepared by dissolving PVA powder (6 g) in 60 mL deionised water containing 6 g of H_2_SO_4_.

### 2.4 Fabrication of symmetrical supercapacitors

Symmetrical supercapacitors were fabricated by sandwiching porous Whatmanfilter paper dipped in the respective electrolytes between two PAg paper electrodes. The electrochemical window, impedance, specific capacitance, energy density, power density, bending test and cycling stability of the fabricated supercapacitors were then evaluated using the cyclic voltammetry, impedance and galvanostatic charge–discharge measurements.

## 3. Characterisation techniques

The surface morphology of the samples coated on paper was studied mainly using various microscopic techniques such as SEM and AFM. Formation of silver nanoparticles formation was studied using TEM analysis. Zeiss EVO 18 cryo-scanning electron microscope (SEM) with variable pressure working at 20–30 kV was used for the SEM analysis. High-resolution transmission electron microscopy (HRTEM) was performed in an FEI TECNAI S Twin microscope with an accelerating voltage of 100 kV. For TEM measurements, the sample solutions were dispersed in the solvent and sonicated well under an ultrasonic vibrator. Then sample solutions were allowed to settle for a while and drop-casted the top solution on a Formvar-coated copper grid using micro pipette and dried at room temperature before observation. Atomic force microscopy (AFM) images were taken using a Bruker Multimode AFM-3COCF (Germany) operating in tapping mode. Particle size measurement of the samples were carried out in a Nano ZS Malvern instrument employing a 4 mW He–Ne laser (*I* = 632.8 nm) and equipped with a thermostated sample chamber. X-ray diffraction studies were performed with an X-ray diffractometer (Philip's X'pert Pro) with Cu-Kα radiation (*I* = 0.154 nm) with the step size and scan rate of 0.02° and 10° per minute respectively employing an X'celerator detector and a monochromator at the diffraction beam side. Films were used by employing a standard sample holder. Cyclic and galvanostatic charge discharge (CV, GCD) studies and impedance analysis were carried out using a CHI6211B electrochemical analyzer, in which flexible paper electrode was used as working electrodes. The specific capacitance could be obtained from the slope of the discharge curve of the galvanostatic charging/discharging mechanism according to eqn.*C*_s_ = *kit*/*m*Δ*v*where *i* is the current, *t* is the elapsed time during the discharge process, Δ*v* is the total working potential, and *m* is the mass of the electrode materials. Commonly, the formula includes the mass of the active materials. *k* is a constant multiplier (*k* = 2 if the mass of a single electrode is used and *k* = 4 if the mass of both electrodes is taken into account). Electrochemical impedance measurements of the device were carried out in the frequency range of 0.1 Hz to 100 kHz at a particular open-circuit potential. Electrical conductivity measurements of the paper electrodes were performed with a standard four-probe conductivity meter using a Keithley 6221 programmable current source and a 2128A nanovoltmeter. UV-vis absorption spectra of the samples were recorded using spectrophotometer (Shimadzu model 2100) in the range of 200–800 nm.

## 4. Results and discussion

### 4.1 Preparation of polyaniline-silver paper electrode (PAg)

**Scheme 1:**
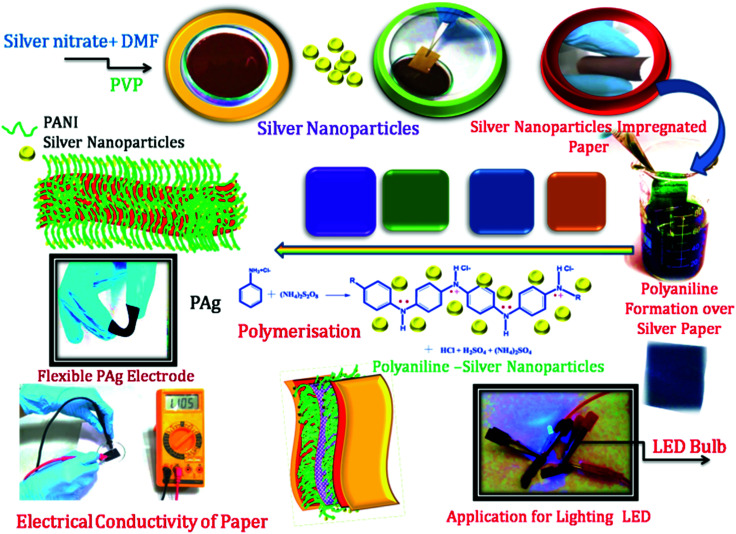
Formation of silver stacked PANI based paper electrodes.

PANI-silver coated paper electrodes (PAg) were prepared by a two-step process involving initial silver nanoparticles coating followed by aniline polymerization over the paper as shown in Scheme 1. PAg paper electrodes were developed with varying amount of aniline by increasing the soaking time of aniline followed by polymerization time using APS as oxidative radical. Silver nanoparticles were prepared from silver nitrate through simple reduction using the solvent DMF in the presence of PVP which provides colloidal stability to the formed nanoparticles. DMF could perform as a reducing agent cum solvent for silver nanoparticles and is known for its superior stability in terms of chemically as well as thermally, and excellent solvent for a wide variety of organic and inorganic materials.^38,39^

**Fig. 1:**
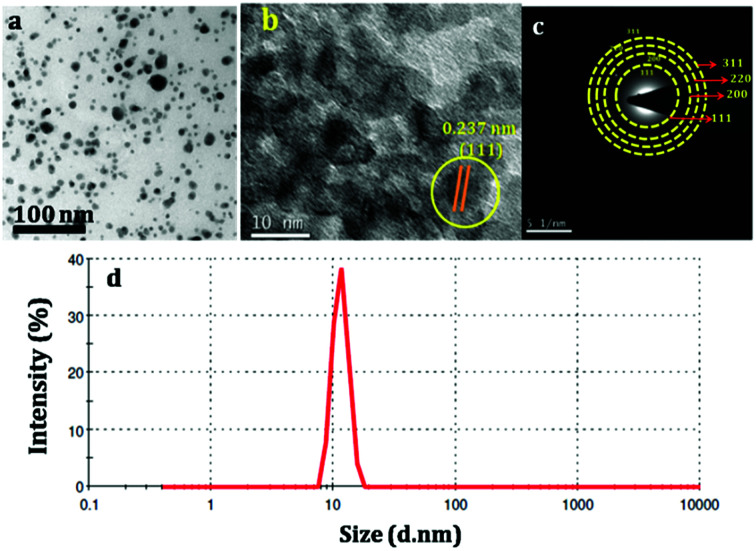
(a and b) TEM and HRTEM of silver nanoparticles, (c) SAED Pattern of silver nanoparticles (d) DLS analysis of silver nanoparticles for particle size measurement.

**Fig. 2:**
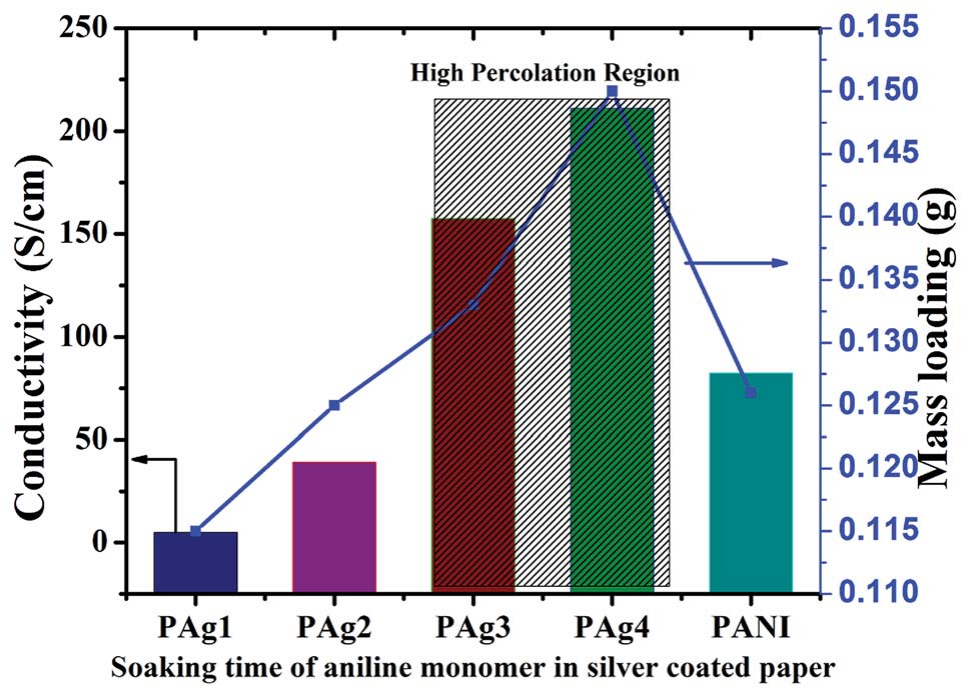
Electrical conductivity of PANI coated on silver paper for different time intervals.

The size of the prepared silver nanoparticles was measured using TEM and particle size analyser (Fig. 1a, b and d, HRTEM is shown in Fig. S1†). TEM analysis confirmed the formation of uniform spherical silver nanoparticles of average size around 10–20 nm, which was further conformed from DLS measurements. HRTEM showed the fringes corresponding to *d* spacing of 0.237 nm which is indexed to 111 plane of silver nanoparticles (Fig. 1b). The prepared nanosilver could be indexed for the face-centered cubic (fcc) structure which was confirmed from SAED (Fig. 1c) and further strengthened from XRD pattern which is being discussed below. PAg paper electrodes were prepared with varying amount of aniline and polymerization time are designated as PAg1, PAg2, PAG3, and PAG4 respectively. The electrical conductivity of PAg films with varying amount of aniline loading and the experimental details are given in the ESI as Table S1.† Electrical conductivities of the prepared PAg's were measured using four probe electrical conductivity meter (Fig. 2). Figure shows the effect of mass loading *vs.* conductivity when moving from PAg1 to PAg4.

The measured conductivity values were found to be increasing from 4.94, 39.23, 157.47, 211.24 S cm^−1^ for PAg1, PAg2, PAg3, and PAg4, respectively. The percolation region was found to be at PAg3 where the conductivity increased drastically beyond which the percolation was achieved.^40^

PAg4 decorated with silver PANI nanowire network exhibited highest conductivity (211.24 S cm^−1^), which is mainly attributed to the uniform silver stacking in the paper thereby providing smooth conducting channels through conductive PANI for efficient electron transport. PANI alone exhibited a conductivity of 82.44 S cm^−1^. The approximate PANI deposition of the PAg4 was measured to be around 0.15 g. The bare paper was weighed to be less than 0.1 g.

The amount of PANI deposited onto the silver paper increased from 0.1 mm to 0.15 mm which corresponds to the uniform coating of PANI upon polymerization for various time intervals. The coating thickness on the silver paper was measured and found to be around 2.25 mm for PAg4. The details are tabulated in Table 1. The conductivity of the PAg electrode at different bending curvature was measured using the four probe conductivity analysis (ESI†). The electrodes were mechanically stable at different bending curvatures and it is measured the surface conductivity (208 and 210 S cm^−1^) at various different bending states, revealing the performance of the PAg electrode is hardly influenced by external stress. PAg4 was optimized as the electrode material for the fabrication of supercapacitor due to its high conductivity.

**Fig. 3:**
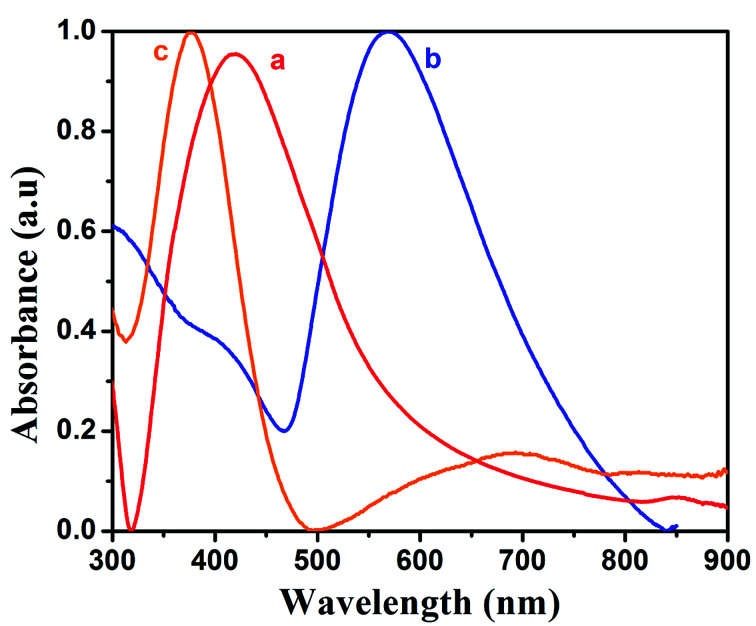
UV-vis spectra of (a) silver, (b) PANI, (c) PAg4.

The opto-electronic properties of the prepared silver nanoparticles, PANI and PAG4 were studied by UV-vis spectroscopy. The UV-vis spectra of pure PANI, Ag and PAg4 nanocomposite are shown in Fig. 3a–c respectively. UV-vis spectra of Ag nanoparticles showed absorption maxima at 400–417 nm corresponding to the surface plasmon resonance energy of the silver nanoparticles. The spectra showed more intense and sharper band which is attributed to the existence of more uniform silver nanoparticles. PANI exhibited spectral maxima at 329 nm and 637 nm associated with π–π* transitions of the benzenoid structure and n–π*transitions of the quinonoid ring, respectively. In comparison with PANI, the peaks at 370 nm and 637 nm are shifted to 390 nm and 750 nm in PAg4 composite, respectively. These changes are related to the presence of more intense polar band generated by the presence of interaction between polyaniline chain and silver nanoparticles. Due to the electrostatic interaction, silver atoms have a large amount of free electrons. These free electrons can easily conjugate with the nitrogen atoms from benzenoid and quinonoid structures of PANI chains. Once the conducting PANI gets impregnated with silver nanoparticles, a conjugating electron cloud between these is formed easily, resulting in a red-shift in UV-vis spectra of PAg nanocomposite. The presence of these formed polarons is expected to for the high conductivity and further enhances the performance of the supercapacitor device.^41–43^

**Fig. 4:**
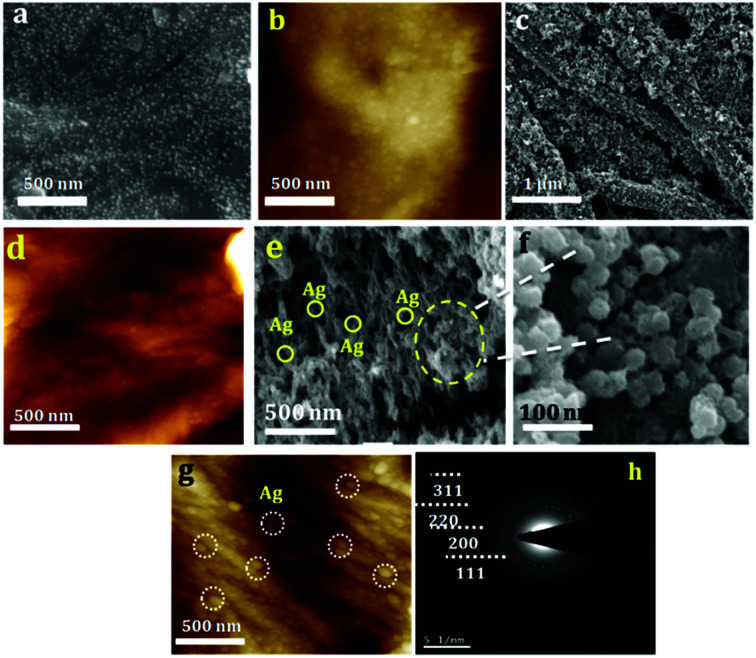
(a and b) SEM and AFM of silver nanoparticles coated on paper, (c and d) SEM and AFM of PANI nanowires coated on paper (e–g) SEM and AFM of PANI nanowires embedded with silver nanoparticles coated on paper (h) SAED pattern of PAg.

Morphology of paper coated with silver nanoparticles, PANI and PAg4 coated on paper were studied by SEM and AFM. Typical SEM and AFM image silver nanoparticles are shown in Fig. 4a and b, respectively. PANI nanowires with a diameter of about ∼100–200 nm were observed to be knotted and wrapped with each other, which leads to the patterning of uniform and highly porous interconnected networks on paper. SEM and AFM of the same are shown Fig. 4c, d and S2,† respectively. The height profile of the same is shown in Fig. S3.† These highly porous PANI network arrangements can drastically improve the supercapacitor performance through efficient uptake of electrolyte, and can also act as an effective pathway in shuttling ion transportation between the PANI and electrolyte. SEM and AFM of PAg4 coated on paper is shown in Fig. 4e–g, respectively. It can be seen that silver nanoparticles are beautifully festooned on the surface of PANI wires. The average diameter of the Ag nanoparticles were about 10–20 nm. The highly monodispersed silver nanoparticles with good dispersion with the polymer matrix is expected to provide a high charge/discharge rate and thus enhancing the specific capacitance of the device. The Ag nanoparticles play a vital role in decreasing the internal resistance and thereby enhancing the electronic conductivity of the paper electrode. Further these Ag nanoparticles can induce the ordering of the PANI chains through the interchain linkage to efficiently bind with nitrogen sites of PANI chains.

The SAED patterns of PAg are shown in Fig. 4h. The concentric rings corresponds to the diffraction pattern of the Ag nanoparticles confirms its polycrystalline nature of the composite. The rings arise because of the reflections from (111), (200), (220), and (311) lattice planes of fcc Ag. Further, crystalline phase and crystallinity of silver particles and PAg were confirmed by XRD.

**Fig. 5:**
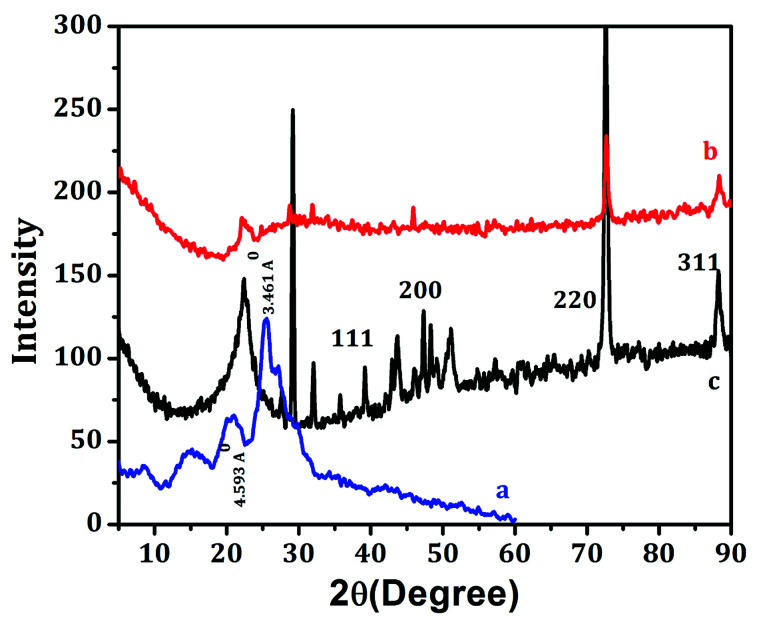
XRD analysis of (a) PANI, (b) Ag nanoparticles, (c) PAg4. All the XRD were taken after coating on the paper.

The XRD patterns of pure Ag, PANI, and PAg4 are shown in Fig. 5. Pure PANI exhibited broad diffractograms in the range of 2*θ* = 10°–30° which is mainly attributed to the parallel and perpendicular periodicity of the PANI chain (Fig. 5a). The broad diffractogram observed at ∼2*θ* = 19.3° and 2*θ* = 25.7° corresponding to the *d* spacing value of 4.593 Å and 3.461 Å, respectively. The broadness of the diffractogram represents the low crystallinity of the PANI which is mainly due to change in the configuration of the repeated of benzenoid and quinoid rings in PANI chain. X-ray diffraction analysis of silver nanoparticles presented distinct peaks corresponding to the lattice planes of fcc of silver nanoparticles that could be indexed for the face-centered-cubic (fcc) structure of silver (Fig. 5b). The peaks positioned at 38.22, 44.35, 64.48, and 77.50° can be related with the (111), (200), (220), and (311) *hkl* values of face-centered-cubic silver nanoparticles. The SAED patterns also showed quite similar, to that of X-ray diffraction patterns which further strengthened the formation of silver nanoparticles. The average crystallite size of the Ag nanoparticle calculated using Scherrer's equation is 25 nm.^44^

For the PAg, the sharp diffractograms at 2θ values of 38.10°, 53.28°, 64.44° corresponded to the face-centered cubic (fcc) phase of silver (111), (142), and (220), respectively (Fig. 5c). The existence of sharp peaks in PAg clearly indicates the presence of fcc crystalline phase of nanoparticles in the nanocomposite with their crystalline nature.

### 4.2 Electrochemical characteristics of the paper based electrodes

The electroactive surface area of the paper electrode was measured using cyclic voltammetry by studying the effect of scan rate as a function of variation in anodic peak current. Fig. S4† shows the plot peak current *vs.* square root of scan rate which showed a linear relationship, indicating that the current linearly increases with the scan rate. The correlation coefficient was found to be 0.990. The electroactive surface area was calculated using Randles–Sevcik equation (eqn (1)) from the plot of peak current *I*_p_*versus* the square root of scan rate,

1 *I*_pa_ = 2.68 × 10^−5^*n*^1/2^*AC*_0_*Dν*^1/2^

where *I*_pa_ represents to the anodic peak current (*A*), *n* is the number of electrons involved, *A* is area of the electrode (cm^2^), *D* is the diffusion coefficient, *C*_0_ is the concentration of electrolyte, and *ν*is the scan rate. The effective electro active surface area can be calculated from the slope of the plot of *I*_pa_*vs. ν*^1/2^ and it was found to be 0.152 cm^2^ for PAg4 electrode. The superior electro-active surface area of the electrode proposes itself as a promising candidate as electrode material which is assumed to provide more efficient electroactive sites.

Flexible supercapacitors were prepared by sandwiching the PANI and PAg4 coated paper electrodes in between the electrolytes bloated filter paper. The electrolytes used are aqueous 1 M H_2_SO_4_ and PVA–H_2_SO_4_ gel electrolytes. The electrochemical window stability of the supercapacitor device (PANI/H_2_SO_4_/PANI and PAg/H_2_SO_4_/PAg) was studied by cyclic voltammetric technique. The extent of the stability of the electrochemical potential window is considered to be very important and there exists a stern concern in aqueous electrolytes where increasing the potential window beyond 1.2 V induces oxygen/hydrogen evolution *i.e.* water decomposition and thereby leading to the damage of the device.

**Fig. 6:**
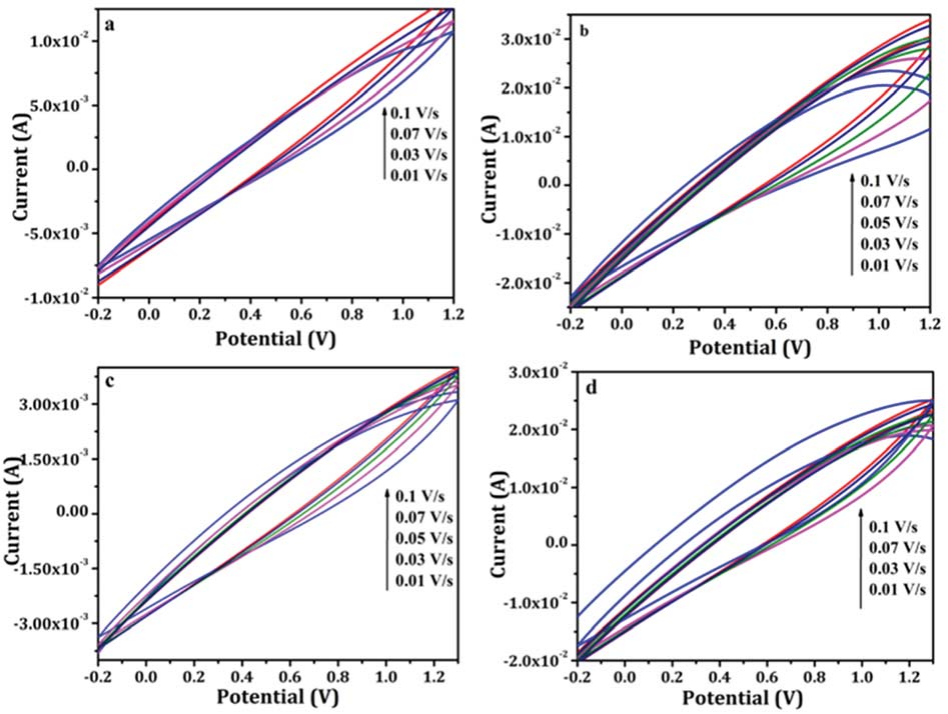
Cyclic voltammograms of (a and b) PANI/1 M H_2_SO_4_/PANI and PAg4/1 M H_2_SO_4_/PAg4 in aqueous electrolyte, (c and d) PANI/PVA–H_2_SO_4_/PANI and PAg4/PVA–H_2_SO_4_/PAg4 in gel electrolyte.

Henceforth, in order to further enhance the potential window, we have measured the performance of the device in gel electrolyte apart from the aqueous electrolyte. In the gel electrolyte system, the potential range was measure upto 1.3 V, in which the paper based supercapacitor system showed an improvement in the device efficiency. Fig. 6a–d shows the cyclic voltammograms measurements recorded using two-electrode system with a voltage window ranging from −0.2 to 1.2 V in aqueous electrolyte and −0.2 to 1.3 V in gel polymer electrolyte. Generally all of the CV exhibited a stable electrochemical voltammograms (−0.2 to 1.2 V) with a hike in the current with increasing scan rates (0.01 to 0.1 V s^−1^). The redox current increased from 0.01 A to 0.03 A in the case of PANI and PAg4 device in aqueous electrolytes (Fig. 6a and b). Similarly in the case of gel electrolyte, PANI and PAg4 devices exhibiting a redox current of 3 mA and 0.03 A respectively (Fig. 6c and d). The increase in the redox current showed the highly conducting nature of PAg4 based electrodes which are mainly related to the uniform dispersion PANI nanowire like network onto the silver paper electrodes thereby further providing more effective surface area that could facilitate for the easy access for the electrolytes. This effect exemplifies the good electrochemical performance of the paper electrodes.^45^

**Fig. 7:**
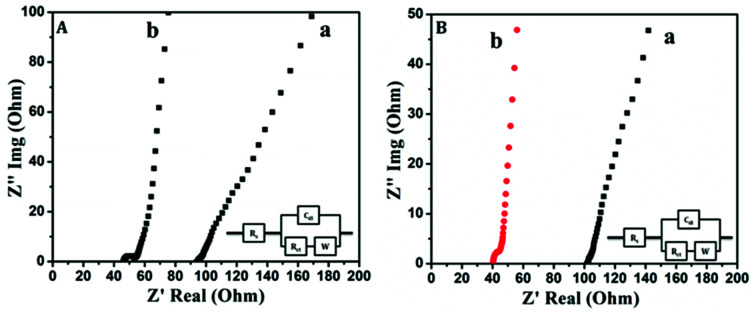
Electrochemical impedance spectra of (A) (a) PANI/1 M H_2_SO_4_/PANI and (b) PAg4/1 M H_2_SO_4_/PAg4 in aqueous electrolyte, (B) (a) PANI/PVA–H_2_SO_4_/PANI and (b) PAg4/PVA–H_2_SO_4_/PAg4 in gel electrolyte. Inset shows the corresponding equivalent circuit diagram.

Electrochemical impedance spectroscopic studies were carried out to analyze the charge transfer mechanism taking place between the electrode and the electrolyte. Fig. 7A and B shows the impedance Nyquist plots of PANI and PAg4 electrode based device in aqueous and gel electrolytes respectively. Electrochemical impedance spectroscopy (EIS) is a non-destructive steady-state technique useful to probe electron transfer and the mechanism of charge conduction process at the electrode/electrolyte interface by applying an oscillating potential as a function of frequency device. The complex impedance (*Z*) can be presented as a sum of the real impedance (*Z*′ (Ω)) and imaginary impedance (*Z*′′ (Ω)), which are components that originate mainly from the resistance and capacitance of the device.*Z* = *Z*′ − *iZ*′′

Typical electrochemical impedance spectrum can be presented in the form of Nyquist plots, *Z*′′ *vs. Z*′ which is recorded as a function of frequency.|*Z*| = [(*Z*′)^2^ + (*Z*′′)^2^]^1/2^

Generally, Nyquist plot includes three regions: a nearly vertical line at low frequencies characterized by capacitive behaviour, a nearly diagonal line at intermediate frequencies related to diffusion resistance, and a semicircle at high frequencies presenting the charge transfer resistance. The device in aqueous electrolytes exhibited a small semicircle followed by a straight line with a *R*_ct_ of 140 Ω and 70 Ω for PANI and PAg respectively representing the characteristics of a diffusion limited electron-transfer process on the electrode surface.^46^ The plot shows a semicircle followed by a Warburg line.

Similarly, the device in gel electrolytes exhibited a similar trend with a *R*_ct_ of 140 Ω and 55 Ω for PANI and PAg4 respectively.^46^ In both the cases the PANI electrode based device exhibited a larger semicircular arc compared to the PAg4 based device which represents an interfacial resistance due to the electrostatic repulsion on the surface of the electrode and electrolyte interface. The plot at the low frequency region was almost vertical close to ∼90° which further suggested that the PAg4 demonstrated a diffusion controlled Warburg capacitive behaviour. This resulted in higher electron transfer resistance for PANI based devices. The inset displays the equivalent circuit (Randles model) used to fit the Nyquist diagrams, which consists of a solution resistance *R*_s_ connected in series with the double layer capacitance (*C*_dl_), charge transfer resistance (*R*_ct_), and Warburg impedance (*Z*_w_), respectively.^40^

The specific capacitance of the supercapacitors at current density of 5 mA g^−1^ were evaluated from the galvanostatic charge/discharge using eqn (2) given below.

**Fig. 8:**
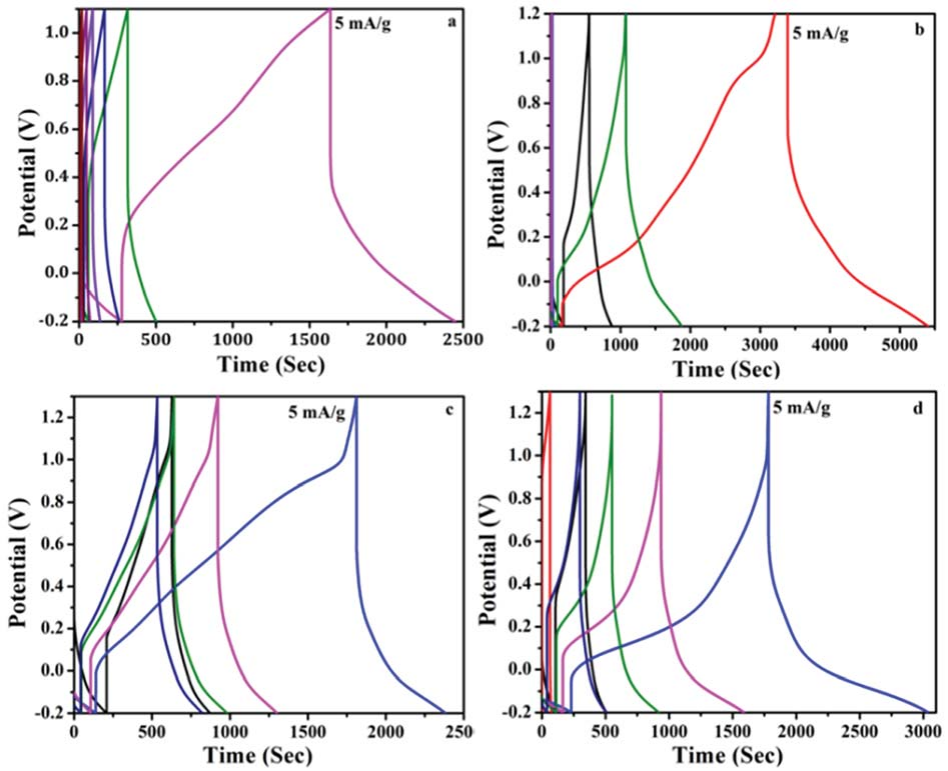
Galvanostatic charge/discharge profiles of (a) PANI/1 M H_2_SO_4_/PANI and (b) PAg4/1 M H_2_SO_4_/PAg4 in aqueous electrolyte, (c) PANI/PVA–H_2_SO_4_/PANI and (d) PAg4/PVA–H_2_SO_4_/PAg4 in gel electrolyte (all the measurements were taken at a current density of 5 mA g^−1^).

2 *C*_s_ = 4*I*Δ*t*/*m*Δ*V*

where *C*_s_ was specific capacitance, *I* is the current (5 mA g^−1^), Δ*t* is the discharge time, Δ*V* is the voltage window (1 V), and *m* is the mass of electroactive material coated onto the electrode.^47–50^Fig. 8a–d shows the charge/discharge profile of PANI and PAg4 based devices in aqueous electrolyte. The stable symmetric charge/discharge curves exhibited by the cells entails that their electrochemical behaviour originates from the typical pseudocapacitance nature of conductive PANI and high electronic properties of the ordered silver nanoparticles.

The details of the various parameters calculated for the device is listed in Table 2. The drop in the internal resistance (IR) for PANI based systems elucidates the presence of internal series resistance, which measured the capacitance as 467 and 213 F g^−1^ in aqueous and gel electrolytes respectively. However PAg4 coated electrodes showed higher specific capacitances of 483 and 613 F g^−1^ in the aqueous and gel electrolytes respectively. In both the cases the discharge current is 5 mA. During the charging and discharging period, the conducting silver nanoparticles facilitates the electron transfer and the pseudo capacitive behaviour of PANI enhances the capacitance overall, which can be attributed to the synergetic effect of the composite electrode. Further, the high conductivity on the surface of the electrode will permit huge number of ions to flow freely after the adsorption of electrolyte by the electrodes, which further facilitates towards the rapid shuttling of the ions in the active species, by providing a smooth pathway through the nanostructured particles for the efficient transport of electrons. The final device weighed about less than 0.5 g which illustrates its very light weight nature, further which can be used for its commercialization.

**Table 2:** Device properties of PANI and PAg4 in 1 M H_2_SO_4_ aqueous and PVA–H_2_SO_4_ gel electrolytes

Sample and electrolyte	Potential (V)	Current (A g^−1^)	Specific capacitance (F g^−1^)	Energy density (W h kg^−1^)	Power density (W kg^−1^)
PANI (1 M H_2_SO_4_)	−0.2 to 1.2	5	467	64.86	130.82
PANI (PVA–H_2_SO_4_)	−0.2 to 1.3	5	213	29.58	119.13
PAg (1 M H_2_SO_4_)	−0.2 to 1.2	5	483	69.56	243.44
PAg (PVA–H_2_SO_4_)	−0.2 to 1.3	5	613	85.13	405.37

The specific energy density and power density were calculated using the eqn (3) and (4).3
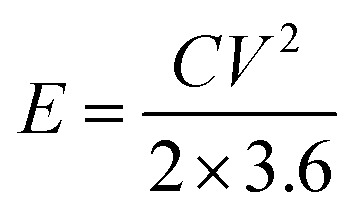
4
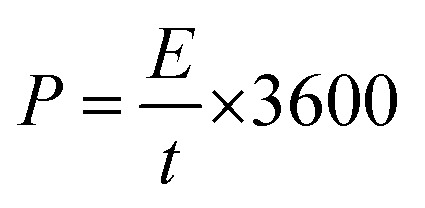


The symmetric supercapacitor device of PANI electrodes revealed an energy density of 64.86 W h kg^−1^ and 29.58 W h kg^−1^ in H_2_SO_4_ and PVA–H_2_SO_4_ respectively while that of device prepared with PAg electrodes showed 67.08 and 85.13 W h kg^−1^ respectively. Similarly the power density of the device with PANI electrodes was measured to be 119.13 and 130.82 W kg^−1^ and that of PAg device exhibited very high power density of 243.44 and 405.375 W kg^−1^ in H_2_SO_4_ and PVA–H_2_SO_4_ electrolytes respectively at 5 mA g^−1^ current density. The higher diffusion conductance of ions through the porous structures is the sole reason for enhancing the power density for PAg based electrodes. This high performance of the device exhibited in solid electrolytes can be endorsed to their exceptional property of the electrodes which exhibited network like nanostructures and its associated high electrical conductivity of PANI networks arising from the silver nanoparticles.

**Fig. 9:**
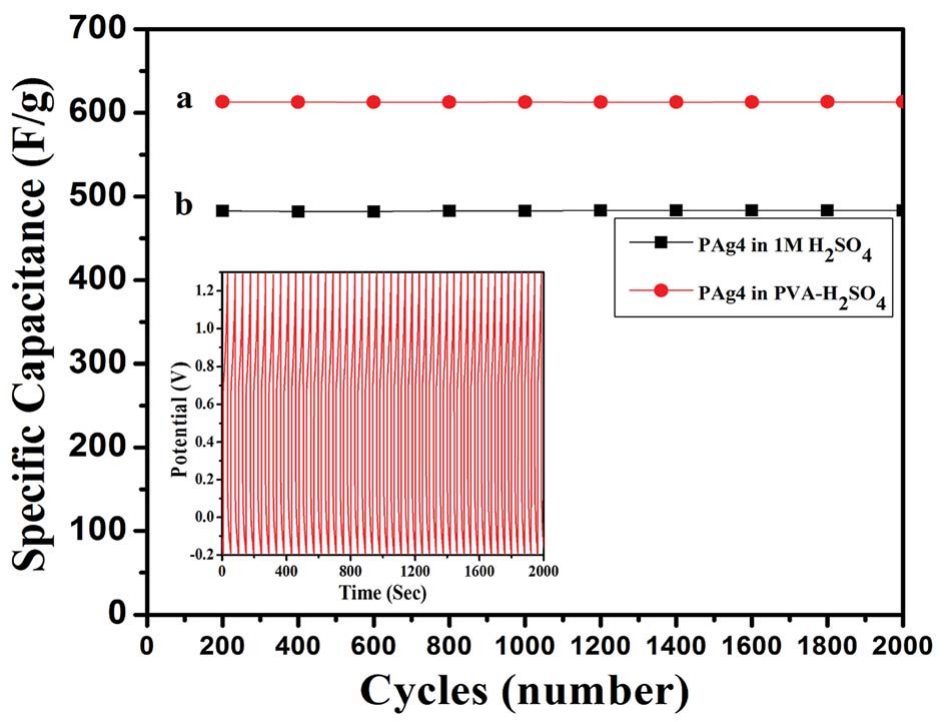
Cyclic stability of (a) PAg4 in 1 M H_2_SO_4_, (b) PAg4 in PVA–H_2_SO_4_.

The cyclic stability of device is a very important property to be evaluated for testing the performance of flexible device. The PAg4 based device was subjected to 2000 cycles for measuring the cyclic stability using GCD. The cell measured about 85% retention in its specific capacitance and the results are shown in Fig. 9.

The measured high specific capacitance of PAg4 based devices in gel electrolyte when compared with its aqueous counterparts is mainly ascribed to the fast and efficient faradic reactions happening in the PANI networks in the presence of highly conducting silver nanoparticles as compared to pure PANI. The reduction in the internal resistance produces charge carriers within the conducting networks due to which is the cells based on PAg4 measured higher performance. A comparison of the performance of various paper based supercapacitor devices is shown in Table 3.

**Table 3:** Comparison of properties of flexible paper based hybrid supercapacitor systems

Electrodes	Methods of preparation of electrode	Specific capacitance	Energy density	Power density	Cycling stability	Reference
Au-PANI/paper	Thermal evaporation/electrodeposition	560 F g^−1^	0.035 W h cm	100 W cm^3^	10 000 cycles	30
Graphene-PANI/paper	Electropolymerization	763 F g^−1^	—	—	1000 cycles	31
Polypyrrole/paper	Chemical oxidative polymerization	0.42 F cm^−2^	1 mW h cm^3^	0.27 W cm^3^	10 000 cycles	23
Silver nanodendrites/cellulose acetate	*In situ* hydrogenation	2.37 F g^−1^	—	—	1000 cycles	51
Graphene-PANI/paper	Hydrothermal/polymerization	464 F g^−1^	—	—	1000 cycles	52
PANI-graphene/Ni foam	Hydrothermal/polymerization	261 F g^−1^	23.3 W h kg^−1^	399 W kg^−1^	1000 cycles	53
Ag-PANI/CNT	*In situ* chemical polymer	528 F g^−1^	87.73 W h kg^−1^	4185 W kg^−1^	1000 cycles	54
RGO/Mno_2_@PANI-graphene	Hydrothermal/polymerization	166 F g^−1^	18.33 W h kg^−1^	0.388 kW kg^−1^	5000 cycles	55
Ni/Co(OH)_2_/NiAc	Electrodeposition	1610/404 mF cm^−2^	0.64 mW h cm^−3^	6 mW cm^−3^	500 cycles	56
Ag-PANI	Chemical reduction/chemical oxidative polymerization	613 F g^−1^	85.13 W h kg^−1^	405.37 W kg^−1^	2000 cycles	Present work

**Fig. 10:**
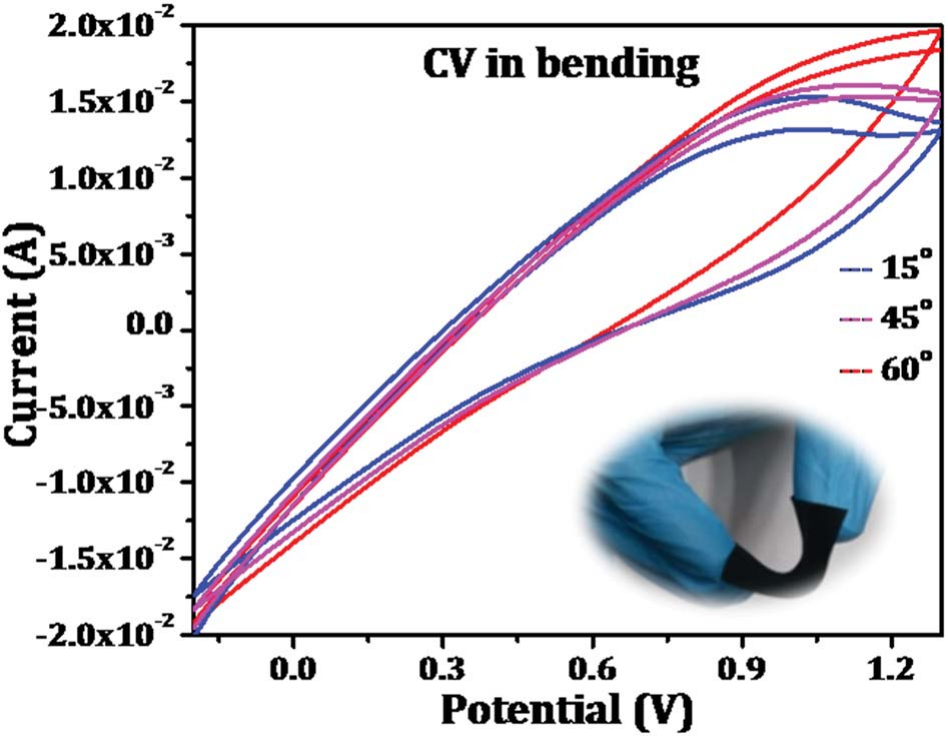
Cyclic voltammetry of PAg in PVA–H_2_SO_4_ under different bending angles.

Further, the mechanical flexibility of the as-fabricated symmetrical supercapacitors was evaluated by making the device through different angles of bending. The results are shown in Fig. 10 which represents the capacitive behaviour of the device was retained which confirms that the flexible device has excellent mechanical deformation capability. All these results evidence its application in flexible electronic gadgets and performances are to be evaluated.

## 5. Conclusion

In conclusion, we have successfully developed a sustainable facile strategy for the fabrication of low cost, highly flexible and high-performance supercapacitors based on silver nanoparticle decorated PANI nanowires coated printing paper electrode. The silver nanoparticles of 10–20 nm decorated with PANI networks based paper electrode measured an electrical conductivity of 211 S cm^−1^. Further fabricated supercapacitor device which showed specific capacitance of 613 F g^−1^, electron density (85.13 W h kg^−1^ and power density 405.37 W kg^−1^) and cycling stability (90% of its capacitance retention even after 2000 cycles). The prepared supercapacitor exploited for lighting LED bulb. All these excellent properties of this sustainable flexible supercapacitor open up new window for the fabrication of flexible and wearable energy storage devices for integrating with flexible electronic gadgets.

## Conflicts of interest

There are no conflicts to declare.

## Supplementary Material

RA-008-C8RA06784H-s001
