# Supplementary material for: Nanowires of polyaniline festooned silver coated paper electrodes for efficient solid-state symmetrical supercapacitors
Source: RSC Adv. 2018 Oct 2;8(58):33314–24. doi: 10.1039/c8ra06784h (PMC9088673; doi:10.1039/c8ra06784h)
Supplement: RA-008-C8RA06784H-s001 [file RA-008-C8RA06784H-s001.pdf]

Electronic Supplementary Material (ESI) for RSC Advances.
This journal is © The Royal Society of Chemistry 2018

## Supporting Information

# NANOWIRES OF POLYANILINE FESTOONED SILVER COATED PAPER ELECTRODES FOR EFFICIENT SOLID- STATE SYMMETRICAL SUPERCAPACITORS

A. Aashish[a,c], C. Molji[a,c], Ganesan Krishna Priya[d], Muthusamy Sankaran[d], Unnikrishnan Nair Saraswathy Hareesh*[b,c], Sudha J. Devaki*[a,c]

a) Chemical Sciences and Technology Division,

b) Materials Science and Technology Division,

c) Academy of Scientific and Innovative Research (CSIR-NIIST Campus), CSIR-National Institute for Interdisciplinary Science and Technology, Trivandrum-695019, India

d) ISRO Satellite Centre, Old Airport Road, Vimanapura Post, Bengaluru-560017, Karnataka, India

| Sample | Soaking in Aniline Monomer (minutes) | Polymerization Time (minutes) |
|---|---|---|
| PAg1 | 5 | 5 |
| PAg2 | 15 | 15 |
| PAg3 | 30 | 30 |
| PAg4 | 60 | 60 |
| PANI | 30 | 30 |

Table S1. Experimental details for the preparation of PAg and PANI electrodes.

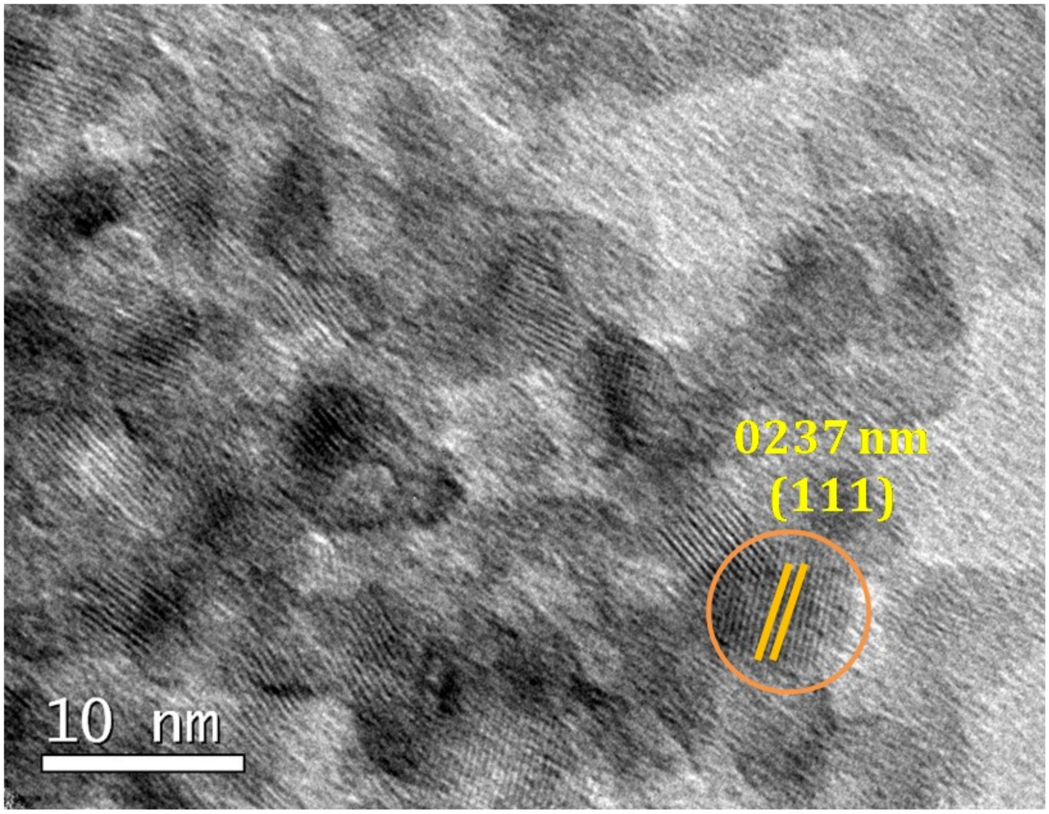


Figure S1. HRTEM of silver nanoparticles.

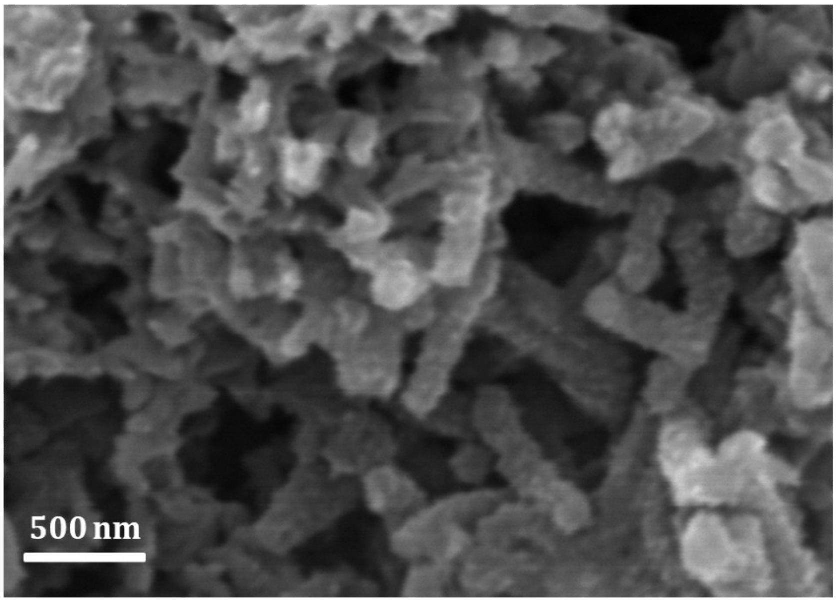


Figure S2. SEM of PANI nanowire like interconnected networks.

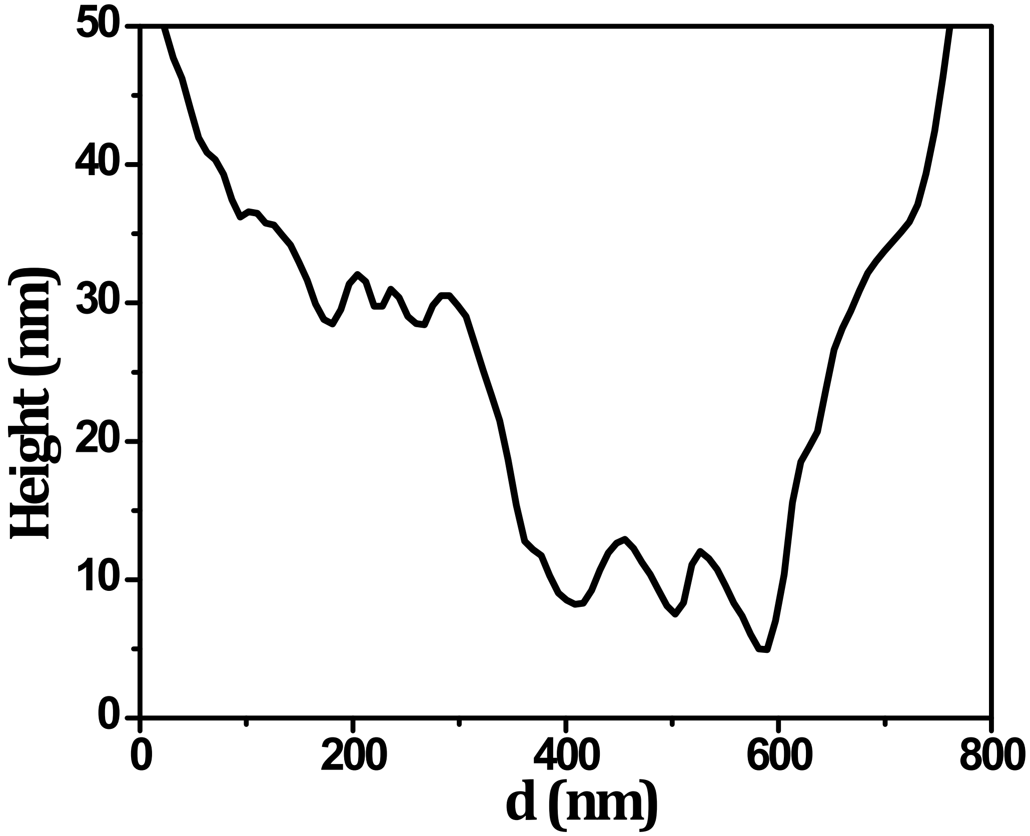


Figure S3. Height profile of AFM.

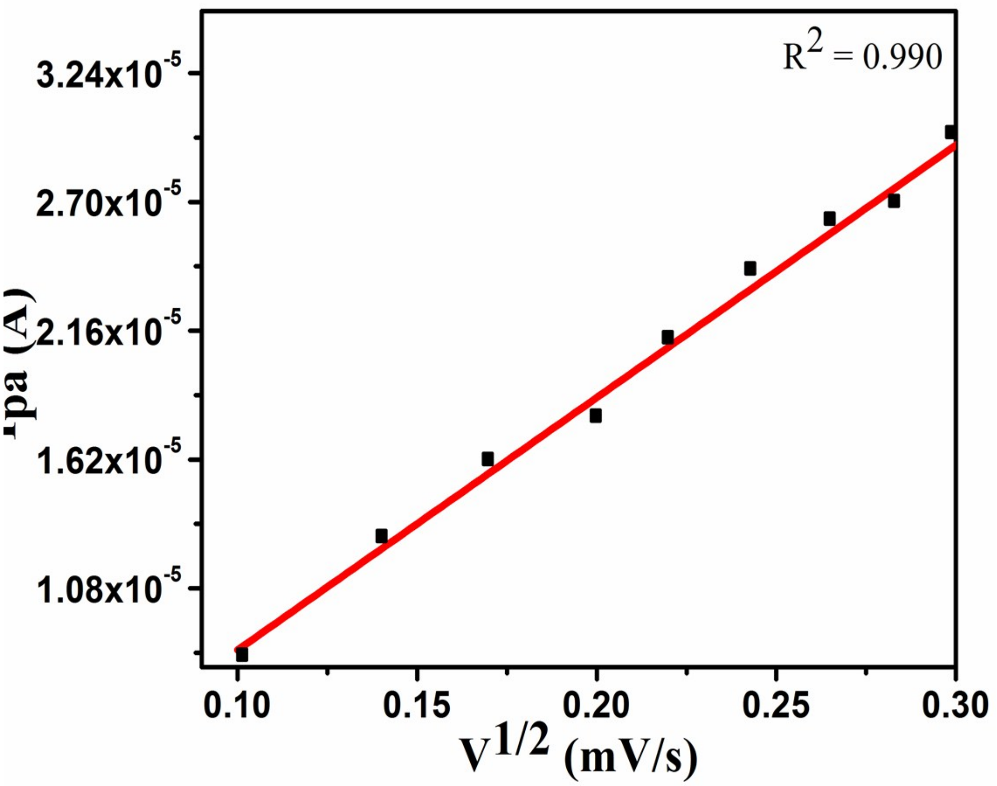


Figure S4. Plot of $I_{pa}$ vs square root of scan rate of PAg4 for calculating the electroactive surface area of PAg4.
